# Progress in Obstetrics and Gynæcology

**Published:** 1898-08-13

**Authors:** 


### PROGRESS IN OBSTETRICS AND GYNÆCOLOGY.

(*Continued from page* 326.)

**Rupture of Uterus.**—Mayo Robson[6] details the necessaries for dealing with a case of ruptured uterus. When operation is indicated and proper appliances are not available, he says that all that is necessary is a stout knitting needle, stout silk or thread, and plenty of towels, handkerchiefs, or other absorbent material, with a piece of elastic tubing, *e.g.*, the tube of an infant's feeding bottle. Should the necessity for immediate operation arise, the above, together with the contents of the pocket surgical case, are boiled, while the patient's skin is disinfected and prepared. The abdomen is then opened, the fœtus extracted if necessary, the uterus brought out through the incision and transfixed with the knitting needle, the elastic ligature passed around the cervix, carefully avoiding the bladder, and the organ removed, the boiled thread or silk being used as suture material. He next describes a case in which abortion occurred, and great difficulty was found in dilating the cervix and removing the ovum, during which process sudden pain and great shock was experienced by the patient. On his arrival he explored the uterine cavity, and found that a rupture had occurred in the posterior wall (this is to be inferred from the context, as it is not precisely stated). He found on making pressure on the abdomen that blood could be forced out of the peritoneal cavity, through the rent. He thereupon curetted the fundus to remove all decidua, and slit up the unimpaired lower portion of the uterus, only to find, however, that the laceration was too extensive to allow of its proper closure by suture. He therefore, by pressure on the abdomen, evacuated as much blood as possible, and then through the rent packed the pouch of Douglas with iodoform gauze, packing the uterus and vagina also with the same material. The packing was repeated once, and the patient made a good recovery.

Purefoy[7] reports two cases occurring at term—one of them was fatal. In the first a full term labour with neglected shoulder presentation occurred, the patient refusing any examination in spite of urgent symptoms until she had been in strong labour for five and a half hours, when a hand was seen protruding from the vulva with the membranes intact. Moulding of the uterus on the body of the child having occurred, decapitation was performed, and delivery effected. Before this, however, it was discovered that the uterus was ruptured on the right side, the intestines being felt with the tips of the fingers. The placenta was extracted by the hand. The rent, which was in the right posterior quarter of the lower segment, was filled with a thick plug of iodoform gauze passed right through it and pressing back the intestines, the lower end of the gauze being in the vagina. The gauze was removed in 24 hours, the patient having been removed to hospital meanwhile. (The delivery took place at the patient's home.) The patient made a good recovery.

The second case[8] occurred in hospital. The patient had been 19 hours in labour when sudden collapse occurred, with rapid and feeble pulse; previous to which her pulse and temperature had both risen, the head still remaining above the brim. Craniotomy was performed and the child delivered. The patient died two days later, and it was found, post-mortem, that she had sustained a laceration of the uterus, partly by contusion, and a laceration of the bladder. The obstruction and contusion were caused by an osseous projection on the posterior aspect of the symphysis, which materially shortened the conjugate diameter and pressed on the advancing head.

**Pregnancy and Labour after Abdominal Myomectomy.**—That pregnancy and normal labour not infrequently result after the enucleation of fibroid tumours of the uterus is well known, but the following case illustrates the possibility of a curious and rare complication after such an operation.

Woyer[9] reports a case in which a woman, aged 34, had been operated on for the removal of a subserous uterine fibroid by abdominal section, followed by the enucleation of the tumour. She made a perfect recovery as far as the immediate results of the operation went, with the exception that an abdominal fistula remained, which evidently communicated with the uterine cavity, since at the menstrual period a few drops of blood exuded from it. After a certain interval the patient became pregnant and miscarried; this was followed by more bleeding through the fistula. A second pregnancy ensued, and the patient went to term, just before which she entered hospital; the fistula now was discharging bloody serum at intervals; this discharge increased very materially on the commencement of labour, and was apparently increased considerably by the movements of the fœtus, and during the course of the labour the membranes bulged through the opening. As labour progressed the size of the fistula increased, until the finger could be passed through it into the uterus, so as to touch and map out the child. Bleeding again occurred towards the end of labour, and meconium was discharged through the fistula, the fœtal heart sounds becoming irregular. Labour terminated naturally, the child being stillborn, and there was no subsequent bleeding from the fistula. The patient made a good recovery, and the fistula closed spontaneously.

**Acute Strangulation of Prolapsed Uterus and Vagina.**—Complete procidentia or extrusion of the entire uterus is a comparatively common affection, but the strangulation of the herniated mass may be regarded as an extremely rare complication. Its rarity is due to the freedom of anastomosis of the uterine vessels and their large number, but when it is remembered to what an extent hæmorrhage from the uterine or ovarian vessels can be checked by twisting the broad ligament it can readily be understood how this accident might be produced.

Baldy and Bryea[10] report each a case of this accident. In Baldy's case the patient was aged 70, and had suffered from complete prolapse for 10 years, which lately had given rise to considerable trouble owing to the difficulty in keeping it up. Nothing was noted on examination beyond the prolapse and some ulceration. Ten days after examination the patient developed serious symptoms with fever, rapid pulse, and vomiting. The prolapsed mass was found on examination to have increased enormously in size, and to present a blackish appearance resembling a strangulated hernia. After considerable difficulty reduction was effected with immediate relief and rapid cessation of the severe symptoms. Under treatment the parts rapidly regained their normal appearance, and at the end of a week abdominal section was performed with the object of amputating the uterus, but owing to the presence of a tumour in the posterior wall total hysterectomy was performed followed by anterior and posterior colporraphy; the patient made a good recovery. The second case, reported by Bryea, was more complicated, from the fact that septic infection had already taken place, gravely affecting the prognosis. The patient was 22 years of age, and had borne four children. Complete prolapse of the uterus and vagina had existed for four years, and had lately become irreducible. The acute symptoms when she was first seen by the writer had lasted a week, and commenced with a rigor which was followed by fever, rapid pulse, and abdominal pain; three days later persistent vomiting set in with constipation. She was found, when Bryea first saw her, to have a temperature of 101, pulse 120, constant vomiting, abdominal tenderness, but no distension; the bowels had not acted for three days, the urine was normal. A swelling, the size of the fœtal head, protruded from the vulva, composed of the prolapsed uterus, vagina, and bladder. The mass could not be reduced. The condition became worse, and three days later, as the mass had a gangrenous appearance—the signs of general septicæmia were present—it was decided to remove the uterus, which was done. At the operation the coils of small intestine were noticed to be reddened, and covered with lymph, showing the existence of peritonitis. Multiple abscesses were found on the uterus, but no abnormality of the appendages. The patient only survived the operation 48 hours. This extremely rare case can only be explained by the occurrence of septic infection in the prolapsed uterus, resulting in septicæmia and peritonitis. The writer alludes to streptococcic infection, but does not state definitely that this micro-organism was actually present, although is is highly probable that it was, and with sanious discharge existence from the cavity and true ulceration of the cervix, which is almost always present in these cases, the avenues for infection are seen to be fairly patent. When also we remember that the streptococcus is actually found in the uterine cavity under conditions leading to no serious symptoms, even locally, it seems surprising that such cases as those described are not more often met with.

**The Bacteriology of Chronic Endometritis.**—Warbasse[11] gives a succinct account of the bacteriology of the vagina. He describes the researches of different writers on the subject, and, on their authority, states that pyogenic streptococci, staphylococci, and diplococci are found in the vaginal secretions of pregnant women. Also he describes the properties of the normal secretion of the vagina, which is acid, and contains one variety of bacillus only, the bacillus vaginæ of Döderlein. The introduction into the normal and healthy vagina of a virgin of staphylococcus aureus, bacillus pyocyaneus and streptococcus was followed by their complete disappearance within four days, the secretion again containing nothing but the bacillus vaginæ. As regards the endometrium, the same micro-organism has been found in so-called healthy cases; but it has also been found that the introduction of streptococci into the cervical canal under ordinary circumstances results in their destruction by phagocytosis. Warbasse removed by a sharp curette from the fundus, in each case, of seventeen women suffering from endometritis a fragment of tissue from which cultures were made. The most careful precautions were taken to guard against the possibility of accidental infection of the culture medium by vaginal or cervical secretion.

Twelve of the specimens showed no growth, the remaining five showed in two cases staphylococcus pyogenes aureus, in one associated with an organism resembling proteus vulgaris, in two cases staphylococcus pyogenes albus, and in one bacterium ureæ.

Warbasse notes that the growth did not take place from the implanted portions of tissue, and is of opinion that under ordinary circumstances these bacilli have no immediate part in the production of chronic endometritis, but that their existence is more or less accidental. He gives several reasons for thinking that their importance is over-estimated. One is that operations on the anus and vagina in which these germs were found showed no febrile reaction; primary union took place in all. Another, that the perforation of the uterus in cases of chronic endometritis, a not unknown accident, is not usually followed by any bad symptoms if sterilised instruments are used. He states, with justice, that we do not know what alterations may occur as the result of gonorrhœal or the more virulent puerperal infection; but that, apart from these, the micro-organisms found have no special significance, and are probably in a state of attenuation and gradual extinction.

[6] Practitioner, July, 1898. [7] Report of Rotunda Hospitals, 1896-97, pp. 6, 7. [8] Loc. cit., pp. 10, 11. [9] Amer. Jour. Med. Sci., Feb., 1898. [10] Med. Chron., Feb. 1898. [11] Amer. Jour. Med. Sci., Feb., 1898.

